# EFFECTS OF AN ART GALLERY INTERVENTION IN PEOPLE LIVING WITH DEMENTIA: A PILOT STUDY

**DOI:** 10.1093/geroni/igz038.204

**Published:** 2019-11-08

**Authors:** Nathan M D'Cunha, Andrew J McKune, Stephen Isbel, Ekavi N Georgousopoulou, Jane Kellett, Nenad Naumovski

**Affiliations:** 1 University of Canberra, Bruce, ACT, Australia; 2 Australian National University, Garran, ACT, Australia

## Abstract

Art gallery programs tailored to the needs of people living with dementia are becoming more popular worldwide. This study aimed to observe the effects of six consecutive weekly discussion-based small group visits to the National Gallery of Australia Art and Dementia program on the salivary cortisol (SC) diurnal rhythm, salivary interleukin-6, quality of life (QoL), depressive symptoms, and cognitive function. Twenty-five participants (17 female; mean age 84.6 ± 7.27 years) completed the study with data collection at baseline, post-intervention, and at a six-week follow-up. Statistical methods were selected based on data distribution. The waking to evening (WE) SC ratio was altered (p = 0.016) (Baseline: 1.35 (1.19, 1.64), Post-intervention: 1.72 (1.54, 1.96), Follow-up: 1.44 (1.22, 1.79)) in the 22 participants who provided viable saliva samples. The WE SC ratio was higher post-intervention compared with baseline (p = 0.011), indicating a more dynamic SC rhythm, but returned to baseline levels at follow-up (p = 0.020). Interleukin-6 levels were unchanged (p = 0.664). In the total sample, no improvements in QoL (Proxy) (p = 0.165) were observed. However, self-reported depressive symptoms differed (p = 0.006), decreasing post-intervention (2.00 (1.00, 2.00)) compared with baseline (3.00 (2.00, 4.50)) (p = 0.015), and verbal fluency was affected (p = 0.027), improving from baseline (2.00 (0.00, 3.00)) to post-intervention (2.00 (0.50, 4.00)) (p = 0.027). Art and Dementia programs appear to have quantifiable benefits, including improved hypothalamic-pituitary-adrenal axis function, justifying a longer controlled trial inclusive of physiological outcomes.

